# A cluster of Psittacosis cases in Lishui, Zhejiang Province, China, in 2021

**DOI:** 10.3389/fcimb.2022.1044984

**Published:** 2022-12-15

**Authors:** Wenwu Yao, Xiuying Chen, Zhuoying Wu, Lingbo Wang, Guoxiang Shi, Zhangnv Yang, Yanjun Zhang, Beibei Wu

**Affiliations:** ^1^ Key laboratory of Vaccine, Prevention and Control of Infectious Disease of Zhejiang Province, Zhejiang Provincial Center for Disease Control and Prevention, Hangzhou, China; ^2^ Department of Microbiology, Lishui Center for Disease Control and Prevention, Lishui, China

**Keywords:** Psittacosis, outbreak, *Chlamydia psittaci*, diagnosis, mNGS

## Abstract

**Introduction:**

Psittacosis, caused by *Chlamydia psittaci*, is widespread throughout the world. In humans, *C. psittaci* infection may lead to severe conditions and complications, including sepsis and multiple organ failure. We report a cluster of cases caused by *C. psittaci* in Zhejiang Province, 2021, which led to one death and three cases of hospitalization.

**Methods:**

The cases were confirmed by nest-PCR, RT-PCR, and mNGS.

**Results:**

The four cases were related and the sequences obtained from the samples were closely correlated with those from Taiwan.

**Discussion:**

This study is the first to report on the case of death from psittacosis in Zhejiang Province, and our results help to assess the disease and recommend effective measures to prevent further spread of *C. psittaci*.

## Introduction

Psittacosis is a zoonotic disease caused by *Chlamydia psittaci* infection. *C. psittaci* is a Gram-negative pathogen that infects humans throughout the world (Yuan et al., 2021). Its infection most commonly occurs in persons with a history of contact with birds or poultry in either occupational settings or companion bird exposure. Over the past 20 years, several *C. psittaci* outbreaks have been reported in different countries with fatality rates of less than 1% (Smith et al., 2005; Mair-Jenkins et al., 2018). The clinical manifestations of psittacosis vary from asymptomatic infection to fatal systemic illness. Humans suffering from symptomatic infection may have headache, chills, fever, malaise, and myalgia (Harkinezhad et al., 2009; Branley et al., 2014; Rybarczyk et al., 2020). Several diagnostic assessments, including serological tests, culture, and PCR, are available (Ménard et al., 2006). With continuous improvement in detection methods in recent years, several cases of *C. psittaci* infection have been confirmed in China using metagenomic next-generation sequencing (mNGS) (Schlaberg et al., 2017; Gu et al., 2019; Chen et al., 2020; Gu et al., 2020).

In this study, we report a cluster of psittacosis cases in Lishui, Zhejiang Province, China, in 2021, which caused one death and three cases of hospitalization. Among the four patients with confirmed psittacosis, three patients belonged to the same family and all four cases were connected. To the best of our knowledge, this study is the first to report on the case of death from psittacosis in Zhejiang Province in recent years, and can help in further research on transmission of *C. psittaci*, drawing attention to psittacosis and strengthening disease surveillance.

## Materials and methods

### Case presentation

**Figure 1:**
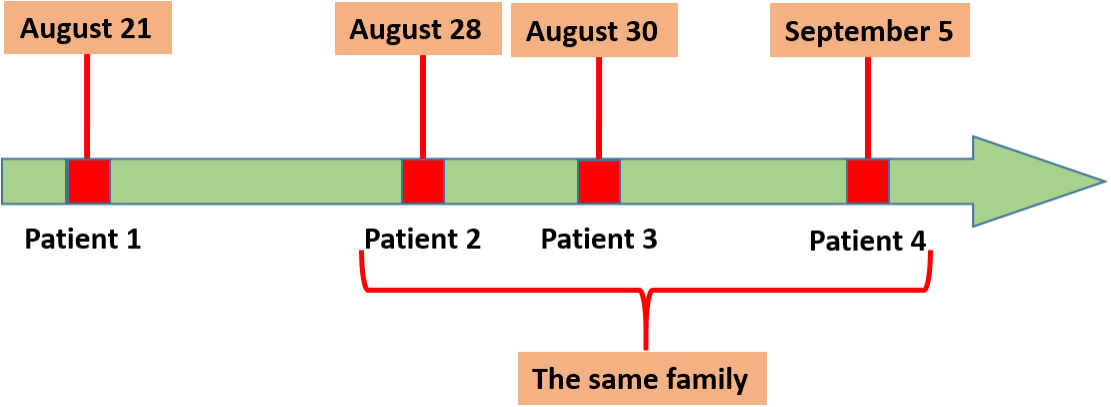
Disease onset in the four patients. Patient 1 was the first to develop symptoms on August 21, 2021, and Patients 2, 3, and4 belong to the same family.

Patient 1 was from Qingyun County and was hospitalized on August 21, 2021, with fever and chills. *C. psittaci* pneumonia was confirmed by the presence of *C. psittaci* sequence reads in his bronchoalveolar lavage fluid and blood samples by mNGS. The other three patients (a farmer, his wife, and son), all from Longquan and with *C. psittaci* infection, were related to Patient 1 and belong to the same family. Patient 2 (65 years old) once bought ducks from Patient 1 and was hospitalized on August 28, 2021, due to severe fever and chills. The condition of Patient 2 deteriorated quickly, and the patient died on September 13, 2021. His wife, Patient 3, developed chills, fatigue, and nausea on August 28, 2021, and was hospitalized for treatment on August 30, 2021. Their son, Patient 4, who attended and stayed with Patients 2 and 3 in the hospital, was hospitalized on September 5, 2021, due to severe fever and chills. The time of disease onset is shown in **Figure 1**.

### Sample collection

Five specimens, including one bronchoalveolar lavage and four serum samples from the four hospitalized patients, were collected from the Central Hospital of Lishui, Zhejiang Province. A total of 74 environmental samples (30 from Patient 1 and 44 from the village of the other three patients), including throat swabs, anal swabs, dung, and drinking water of ducks, chicks, or geese were collected from the patients’ homes.

### DNA extraction and detection

The total DNA of the samples was extracted using a commercial kit (Qiagen, USA), according to the manufacturer’s instructions. The DNA was analyzed by real-time PCR targeting the *C. psittaci* locus tag 16S rRNA and nested PCR (performed at Zhejiang Provincial Center for Disease Control and Prevention (CDC)). The results of nested PCR were confirmed by sequencing at Sangon Biotech Company (Shanghai, China) and BLAST (http://blast.ncbi.nlm.nih.gov/).

### Phylogenetic tree

A total of 11 sequences (ZJLS001-011) were successfully obtained from 15 C*. psittaci*-positive samples by sequencing the nested PCR products. The primers and probes used are displayed in **Table 1**. Phylogenetic analysis based on the 11 sequences and 53 reference sequences from the NCBI GenBank database was performed by using neighbor-joining method with the Tajima-Nei model in MEGA version 6.06, and bootstrap values ≥70% were calculated from 1000 replicates. The reference sequences were chosen from the published ompA sequences, and genotyping was performed as previously described, the information of reference sequences was shown in **Supplement Table S1**.

**Table 1:** Primers used for the detection of *C. psittaci*.

Primer name	Sequence 5’-3’	Amplicon size(bp)
Nest-1-F	GCTACGGGTTCCGCTCT	1017
Nest-1-R	GCTTCGATTCAGATCAACAAA	
Nest-2-F	CGCTCTCTCCTTACAAGCC	1000
Nest-2-R	CAAATTGCTTCGATTCAGATC	
Realtime-F	CACTATGTGGGAAGGTGCTTCA	76
Realtime-R	CTGCGCGGATGCTAATGG	
Realtime-pb	FAM- CGCTACTTGGTGTGAC-BHQ1	

## Results

### Geographical distribution of patients

**Figure 2:**
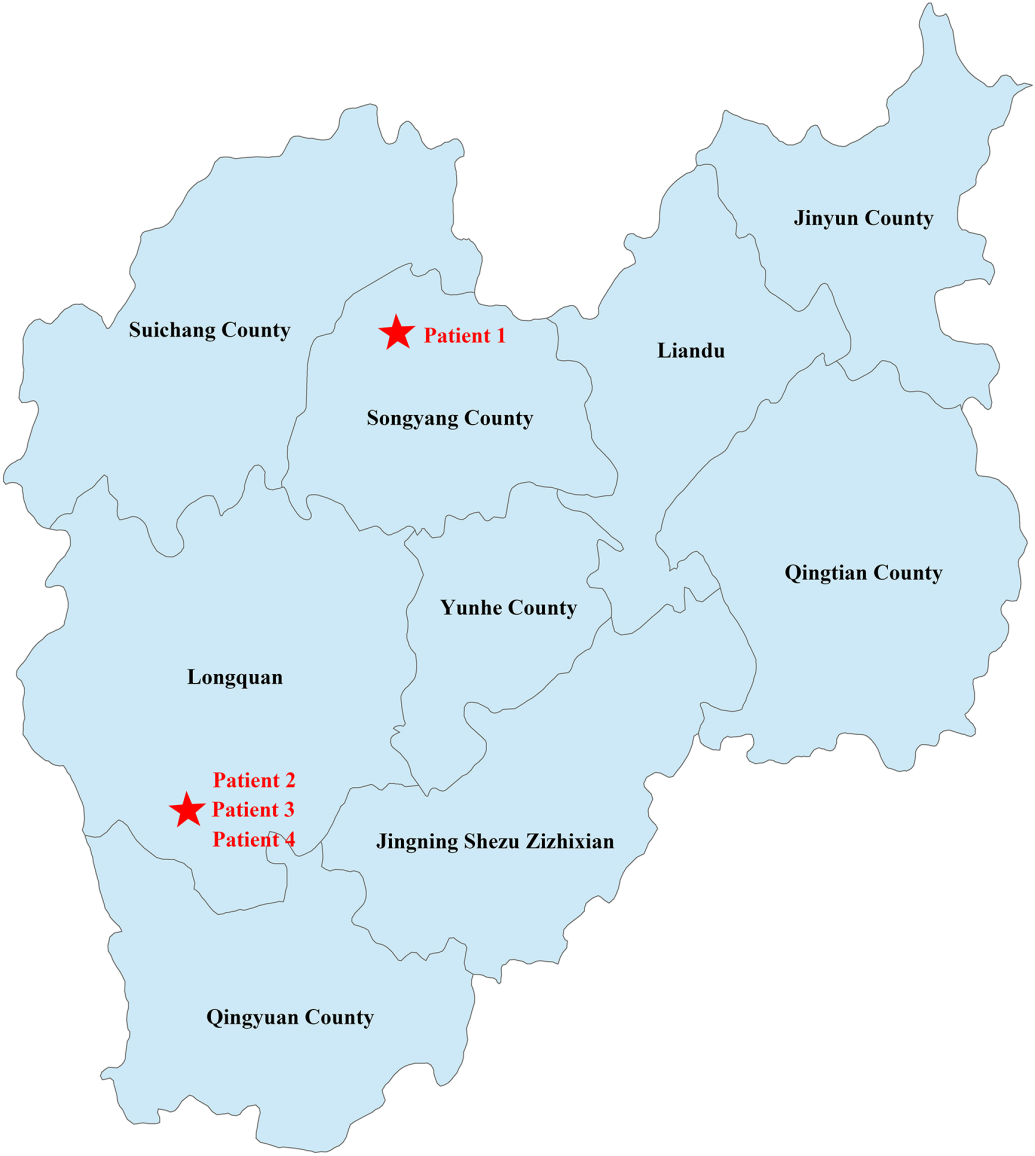
Geographical distribution of the cases. The geographic location of the family has been marked with a red five-pointed star.

Patient 1 was from Qingyun County, while the other three patients were from Longquan, the neighboring county of Patient 1. As shown in **Figure 2**, the two locations are about 80 km apart. Patient 1 was a tranter and had come to the village of the other three patients to sell poultry and Patient 2 had once bought ducks from him.

### Metagenomic next-generation sequencing

**Figure 3:**
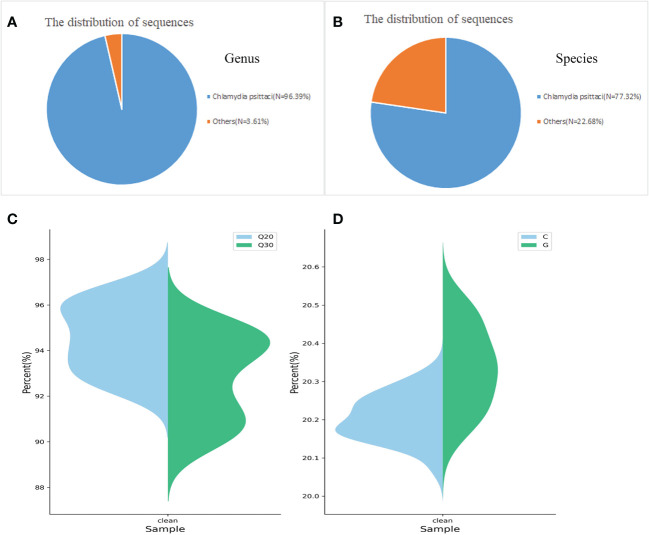
Diagnosis of *C. psittaci* in bronchoalveolar lavage fluid from Patient 1 using mNGS. A total of 8905 and 7144 sequence reads corresponded to *C. psittaci* at the genus and species levels, respectively **(A, B)**. Q20/Q30 distribution **(C)** and G/C distribution **(D)**.

Patient 1 was hospitalized with unexplained fever and chills, and after 3 days, his bronchoalveolar lavage was sent to MATRIDX (Hangzhou, China) for mNGS. The mNGS results revealed that 8905 and 7144 sequence reads corresponding to *C. psittaci* accounted for 96.39% and 77.32% of microbial reads at the genus and species levels, respectively (**Figures 3A, B**). The result of sequencing quality was shown in **Figures 3C, D**. The result of sequencing quality was shown in **Figures 3C, D**.

### Laboratory diagnosis by real-time PCR

All four patients tested positive for *C. psittaci* in RT-PCR. In addition, two environmental samples (dung and drinking water of goose) from Patient 1 and six environmental samples (three throat swabs, two fecal, and one drinking water of duck) from the other three patients (belonging to the same family) were positive for *C. psittaci* (**Table 2**). More importantly, seven environmental samples from the same village of the three patients were positive for *C. psittaci*, indicating widespread transmission of *C. psittaci* among poultry in that village and further potential emergence and spread of psittacosis in Lishui.

**Table 2:** C*. psittaci* positive numbers of environmental samples from four patients.

Patient number	Age	Gender	Throat swabs ofpoultry	Anal swabs of poultry	dung	drinking water of poultry	Total
Patient 1	70	Male	\	\	1	1	2
Patient 2	65	Male	3	2	6	2	13
Patient 3	63	Female					
Patient 4	42	Male					

### Nested PCR

**Figure 4:**
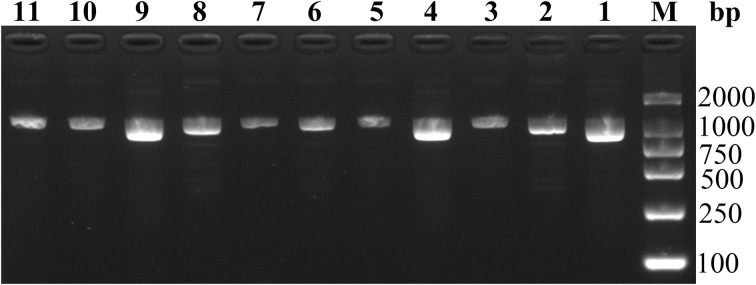
Agarose gel electrophoresis of 11 nested PCR products. M: DL2000 DNA marker, lane 1-11: 1–11 sequences of samples.

A total of 15 C*. psittaci*-positive RT-PCR samples were subjected to nested PCR, and the products obtained were confirmed by 2% agarose gel electrophoresis (**Figure 4**). The results revealed 11 electrophoretic bands of 1000-bp size, and 11 sequences (ZJLS001-011) were successfully obtained by sequencing the nested PCR products.

### Phylogenetic analysis

**Figure 5:**
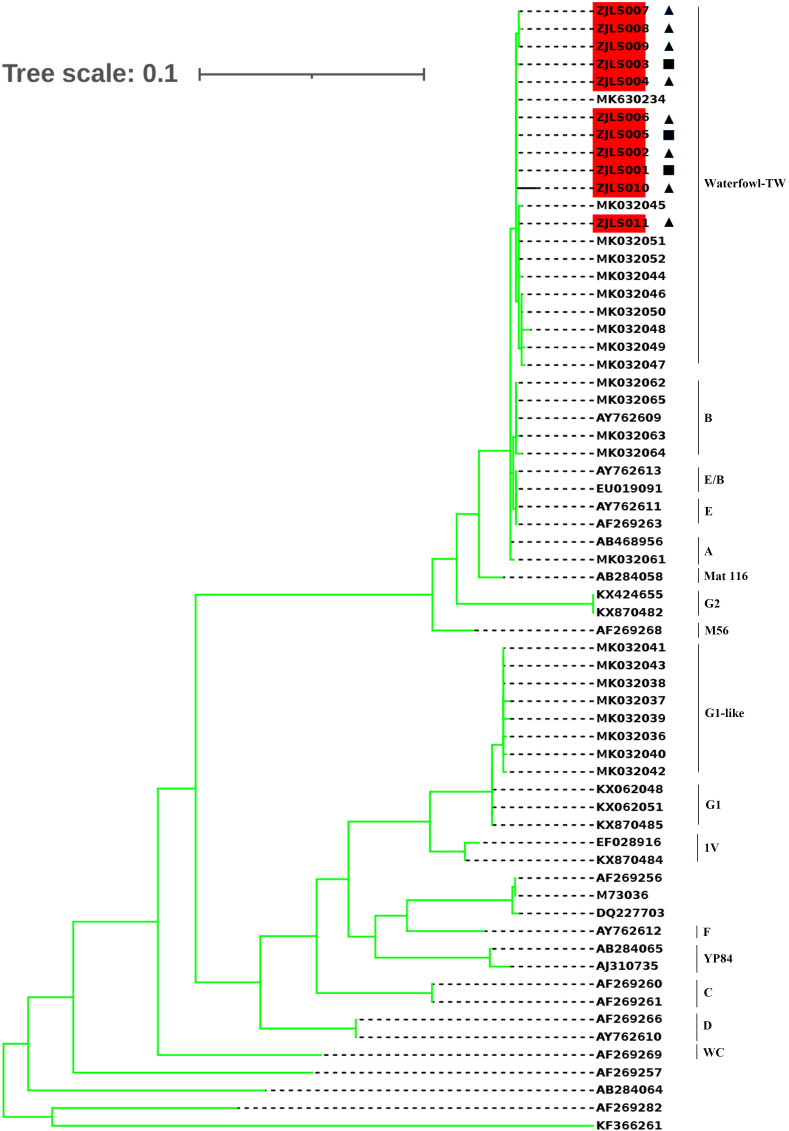
Phylogenetic tree of *C. psittaci* sequences. The 11 sample sequences which are highlighted and 53 reference sequences (the genotype was labled on the right)were compared using Molecular Evolutionary Genetics Analysis (MEGA) software version 7.0 (maximum likelihood phylogeny test) and gamma-distributed rates among sites with 1000 bootstrap replicates. The reference sequences used to construct distinct phylogenetic branches were obtained from GenBank sequence database. ◼Samples collected from Patient 1. ▲Samples collected from Patient 2, 3 and 4.

A phylogenetic tree (**Figure 5**) was constructed based on 11 sample sequences and 53 reference sequences. Phylogenetic analysis showed that the 11 sample sequences were clustered in the same branch and were closely related to MK630234 which is reported in Guangzhou and MK032045 which is from Taiwan, the genotype is waterfowl-TW.

## Discussion

Zhejiang Province is located in eastern China and has a humid subtropical monsoon climate. Mountains and hills account for 74.63% of the total area, with forest coverage rate reaching 60.5%, which is among the highest in China (Yan et al., 2018). The ecological environment of Zhejiang Province provides a suitable habitat for birds, with about 300–400 species of birds inhabiting this area. However, *C. psittaci* infection most commonly occurs in people with a history of contact with birds or poultry in either occupational settings or companion bird exposure. *C. psittaci* infection has been reported worldwide, including China, USA, Europe, and Australia (Yung and Grayson, 1988; Cheng et al., 2013; Branley et al., 2014; Shaw et al., 2019). To the best of our knowledge, the present study is the first to report a cluster of psittacosis cases and a resultant death in Lishui City.

The results of phylogenetic analysis indicated that 11 sequences were almost clustered in the same branch and have high homology. Patient 2 once bought ducks from Patient 1, and the disease onset in the four patients occurred in chronological order, with Patients 1, 2, 3, and 4 hospitalized on August 21, August 28, August 30, and September 5, respectively. The sick duck might have first spread *C. psittaci* to Patient 1, and then Patients 2–4 might have been infected by sick ducks bought from Patient 1. The sample sequences were closely related to MK630234 and MK032045. These findings suggest that *C. psittaci* may infect humans in different regions *via* bird migration.

Patients 2, 3, and 4 belong to the same family, with the father presenting clinical symptoms first, followed by the mother and their son. However, there are rarely any reports on human-to-human transmission of *C. psittaci*, and the reason for *C. psittaci* infection in the mother and son needs further investigation. Patient 1 was found to be positive for *C. psittaci* infection by using mNGS in hospital, and the infection was finally confirmed by RT-PCR and nested PCR in Zhejiang CDC. It must be noted that mNGS requires expensive instruments and professional technicians. Doctors experienced in the treatment of *C. psittaci* infection are also important. Moreover, the government and health administration should expand *C. psittaci* surveillance, and clinicians evaluating fever of unknown origin should consider psittacosis as a possible diagnosis. The Lishui Government quickly organized control measures, including harmless treatment of poultry in the patients’ villages, thorough disinfection of the environment, and increasing public awareness about *C. psittaci* infection.

## Data availability statement

The original contributions presented in the study are included in the article/**Supplementary Material**. Further inquiries can be directed to the corresponding authors.

## Ethics statement

Written informed consent was obtained from the individual(s) for the publication of any potentially identifiable images or data included in this article.

## Author contributions

Methodology: ZW. Formal analysis: GS. Writing—original draft preparation: LW. Writing—review and editing: WY, XC. Supervision: ZY. Project administration: YZ, BW. All authors have read and agreed to the published version of the manuscript. All authors contributed to the article and approved the submitted version.
