# Supplementary material for: A cluster of Psittacosis cases in Lishui, Zhejiang Province, China, in 2021
Source: Front Cell Infect Microbiol. 2022 Dec 15;12:1044984. doi: 10.3389/fcimb.2022.1044984 (PMC9798449; doi:10.3389/fcimb.2022.1044984)
Supplement: Supplementary file 1 [file Table_1.docx]

# Supplement table S1. The sequence information used for the phylogenetic analysis

| Strain | Genbank accession number | region | year |
| --- | --- | --- | --- |
| Daruma | AB284065 | Japan | 1981 |
| B577 | M73036 |  | 1993 |
| / | AF269256 | USA | 2001 |
| FP Baker | AF269257 | USA | 2001 |
| CT1 | AF269260 | USA | 2001 |
| NJ1 | AF269266 | USA | 2001 |
| MNRh | AF269263 | USA | 2001 |
| GPIC | AB284064 | USA | 2001 |
| M56 | AF269268 | USA | 2001 |
| WC | AF269269 | USA | 2001 |
| / | AF269282 | USA | 2001 |
| 84/2334 | AJ310735 | Belgium | 2003 |
| 7344/2 | AY762610 | Belgium | 2005 |
| 7778B15 | AY762612 | Belgium | 2005 |
| 41A12 | AY762609 | Belgium | 2005 |
| 3759/2 | AY762611 | Belgium | 2005 |
| WS/RT/E30 | AY762613 | Belgium | 2005 |
| / | DQ227703 | Taiwan(China) | 2005 |
| Mat116 | AB284058 | Japan | 2006 |
| 1V RUSSIA 2007 | EF028916 | Russia | 2007 |
| VG65-22-3 | EU019091 | GERMANY | 2008 |
| / | AB468956 | Japan | 2008 |
| CPX0308 | AB284064 | Jappan | 2009 |
| 10DC88 | KF366261 | Germany | 2014 |
| 14-95/6 | KX062048 | Poland | 2016 |
| 14-106/3 | KX062051 | Poland | 2016 |
| 15-58d/44 | KX870484 | Poland | 2016 |
| 15-49d/3 | KX424655 | Poland | 2016 |
| 15-48d/9 | KX870482 | Poland | 2017 |
| 15-70d/24 | KX870485 | Poland | 2017 |
| SZ15 | MK630234 | Guangzhou(China) | 2019 |
| GCH03 | MK032036 | Taiwan(China) | 2019 |
| MDNT02 | MK032037 | Taiwan(China) | 2019 |
| MDHL04 | MK032038 | Taiwan(China) | 2019 |
| MDYL09A | MK032039 | Taiwan(China) | 2019 |
| MDPT01 | MK032040 | Taiwan(China) | 2019 |
| MDTN03 | MK032041 | Taiwan(China) | 2019 |
| MDPT06A | MK032042 | Taiwan(China) | 2019 |
| MDKS11 | MK032043 | Taiwan(China) | 2019 |
| GTC01B | MK032044 | Taiwan(China) | 2019 |
| GTC02 | MK032045 | Taiwan(China) | 2019 |
| MDHL05 | MK032046 | Taiwan(China) | 2019 |
| MDHL07 | MK032047 | Taiwan(China) | 2019 |
| MDYL09B | MK032048 | Taiwan(China) | 2019 |
| MDPT06B | MK032049 | Taiwan(China) | 2019 |
| MDPT08 | MK032050 | Taiwan(China) | 2019 |
| MDKS10 | MK032051 | Taiwan(China) | 2019 |
| MDTN12 | MK032052 | Taiwan(China) | 2019 |
| PAPM0724 | MK032061 | Taiwan(China) | 2019 |
| PICL0305 | MK032062 | Taiwan(China) | 2019 |
| PICL0913 | MK032063 | Taiwan(China) | 2019 |
| PICL0503 | MK032064 | Taiwan(China) | 2019 |
| PIST0706 | MK032065 | Taiwan(China) | 2019 |
| ZJLS001 | / | Lishui(China) | 2021 |
| ZJLS002 | / | Lishui(China) | 2021 |
| ZJLS003 | / | Lishui(China) | 2021 |
| ZJLS004 | / | Lishui(China) | 2021 |
| ZJLS005 | / | Lishui(China) | 2021 |
| ZJLS006 | / | Lishui(China) | 2021 |
| ZJLS007 | / | Lishui(China) | 2021 |
| ZJLS008 | / | Lishui(China) | 2021 |
| ZJLS009 | / | Lishui(China) | 2021 |
| ZJLS0010 | / | Lishui(China) | 2021 |
| ZJLS0011 | / | Lishui(China) | 2021 |

1.The accession numbers and origin of the GenBank samples are provided
